# IS THE WAIST/HEIGHT RATIO A BETTER PARAMETER THAN BMI IN DETERMINING THE CARDIOMETABOLIC RISK PROFILE OF OBESE PEOPLE?

**DOI:** 10.1590/0102-672020210003e1610

**Published:** 2022-01-05

**Authors:** Andressa Bressan MALAFAIA, Paulo Afonso Nunes NASSIF, Ricardo Wallace das Chagas LUCAS, Rodrigo Ferreira GARCIA, José Guilherme Agner RIBEIRO, Laura Brandão DE PROENÇA, Maria Eduarda MATTOS, Bruno Luiz ARIEDE

**Affiliations:** 1 Mackenzie Evangelical Faculty of Paraná, Curitiba, PR, Brazil; 2 University Evangelical Mackenzie Hospital, Curitiba, PR, Brazil; 3 Paulo Nassif Institute, Curitiba, PR, Brazil

**Keywords:** Obesity, Metabolic Syndrome, Diabetes Mellitus, type 2, Anastomosis, Roux-en-Y, Obesidade, Síndrome Metabólica, Diabete Melito tipo 2, Anastomose em Y-de-Roux

## Abstract

**Background:**

The increased prevalence of obesity has led to a significant increase in the occurrence of metabolic syndrome, a recognized risk factor for increased morbidity and mortality from cardiovascular diseases. Hyperglycemia or type 2 diabetes mellitus, dyslipidemia and arterial hypertension are its main components. Since 2015, international guidelines have recognized the benefits of bariatric surgery in each isolated factor of this syndrome.

**Aim:**

To evaluate the impact of Roux-en-Y gastric bypass in this syndrome comparing pre- and postoperative periods with laboratory analysis and to compare waist/height ratio and BMI in relation to the determination of the cardiometabolic risk profile.

**Methods:**

A retrospective study was carried out, selecting 80 patients undergoing Roux-en-Y gastric bypass. Total cholesterol, HDL, LDL, triglycerides, fasting glucose, glycated hemoglobin, insulin, body mass index (BMI), vitamin D, vitamin B12, waist circumference and waist/height ratio in three periods were analyzed: the preoperative period from 1 to 6 months, postoperative from 1 to 6 months and postoperative from 1 to 2 years.

**Results:**

There was an improvement in all parameters of the clinical analyses. The preoperative BMI had a mean value of 39.8, in the preoperative period from 1 to 6 months, the values ​​dropped to 33.2 and in the postoperative period of 1 year, the mean was 26. The perimeter mean values ​​of 118.5 preoperatively, 105.2 postoperatively from 1 to 6 months and 90.3 postoperatively from 1 to 2 years. Waist/height ratio was 0.73, 0.65 and 0.56 in pre, post 1 to 6 months and 1 to 2 years respectively.

**Conclusion:**

Roux-en-Y gastric bypass improves metabolic syndrome and waist-to-height ratio is superior to BMI in the assessment of the cardiometabolic risk profile.

## RESUMO

**Racional:**

O aumento da prevalência da obesidade levou ao aumento significativo da ocorrência de síndrome metabólica, fator de risco reconhecido para aumento da morbimortalidade por doenças cardiovasculares. A hiperglicemia ou diabetes mellitus do tipo 2, dislipidemia e hipertensão arterial são seus principais componentes. Desde 2015, diretrizes internacionais reconheceram os benefícios da cirurgia bariátrica em cada fator isolado desta síndrome.

**Objetivos:**

Avaliar o impacto do bypass gástrico em Y-de-Roux nesta síndrome comparando períodos pré e pós-operatório com análise laboratorial, e comparar a razão cintura/estatura e o IMC em relação a determinação do perfil de risco cardiometabólico.

**Métodos:**

Realizou-se um estudo retrospectivo com base prospectiva selecionando 80 pacientes submetidos à bypass gástrico em Y-de-Roux. Foram analisados o colesterol total, HDL, LDL, triglicerídeos, glicemia de jejum, hemoglobina glicada, insulina, índice de massa corpórea (IMC), vitamina D, vitamina B12, perímetro abdominal e relação cintura/estatura em três períodos: o pré-operatório de 1 a 6 meses, pós-operatório de 1 a 6 meses e pós-operatório de 1 a 2 anos.

**Resultados:**

Houve melhora em todos os parâmetros das análises clínicas. O IMC, no pré-operatório, teve a média dos valores de 39,8, no pré-operatório de 1 a 6 meses, os valores caíram para 33,2 e no pós-operatório de 1 ano média foi de 26. O perímetro abdominal teve média dos valores de 118,5, no pré-operatório, 105,2 no pós-operatório de 1 a 6 meses e 90,3 no pós-operatório de 1 a 2 anos. A relação cintura/estatura teve 0,73, 0,65 e 0,56 no pré, pós 1 a 6 meses e 1 a 2 anos respectivamente.

**Conclusão:**

O bypass gástrico em Y-de-Roux melhora a síndrome metabólica e a relação cintura/estatura é superior ao IMC na avaliação do perfil do risco cardiometabólico.

## INTRODUCTION

The increase in the obese population in the world is exponential, it is estimated that 30% of people are overweight or obese, significantly increasing morbidity and mortality from cardiovascular, oncological, endocrine and liver diseases, among others^11^ ^,^ ^38^ ^,^ ^41^.

Obesity, particularly abdominal obesity, is associated with resistance to the effects of insulin on the peripheral use of glucose and fatty acids, one of the components of the physiopathogenesis of type 2 diabetes mellitus, hyperinsulinemia and the increase in adipocyte cytokines. All these factors significantly increase cardiovascular risk, either alone or in combination. In addition to type 2 diabetes mellitus, other obesity-associated comorbidities, such as hypertension and dyslipidemia, are also direct risk factors for the development of cardiovascular disease. In this context, in 2014, Samson et al.^40^ called the concomitance of these comorbidities as syndrome X, currently known as metabolic syndrome (MS)^9^ ^,^ ^40^.

MS is characterized by abdominal perimeter greater than or equal to 102 cm in men and 88 cm in women; fasting glucose greater than 100 mg/dl; triglycerides above 150 mg/dl; HDL cholesterol less than 40 mg/dl in men and less than 50 mg/dl in women; and arterial hypertension (>130 mmHg, > 85mmHg). It is believed that obesity and insulin resistance are the main factors for the development of this syndrome^17^.

Due to the need for a more effective treatment, the term “metabolic surgery” emerged from the recognition of the metabolic effects of bariatric surgery, in addition to weight loss. Currently, the most performed procedures are Roux-en-Y gastric bypass (RYGB) and sleeve gastrectomy. Most patients with MS obtain significant improvements with bariatric surgery^5^.

A recent review by Hwuang^17^ exploring ideal waist-to-height ratios and subsequent comments^6^ ^,^ ^32^ concluded that height-adjusted waist circumference (known as waist circumference index) is superior to BMI in its association with body fat. This conclusion contrasts with the recent IAC and ICCR (International Atherosclerosis Society and International Chair On Cardiometabolic Risk) Consensus report on visceral obesity, which argued that waist circumference thresholds alone are adequate for the assessment of abdominal obesity in clinical practice^3^ ^,^ ^36^.

There is an unmet need to promote consistent and universal public health message that visceral/central/abdominal obesity is associated with adverse health outcomes^32^. The authors of this research have used the waist-to-height ratio (WHtR) for almost 25 years as an adjunct indicator to BMI. It is a better predictor for central obesity, and superior for cardiovascular risk factors^2^. But the waist circumference index is superior to the WHtR in this respect.

The National Institute of Excellence in Health and Care - NICE - recognized the value of WHtR as an indicator of initial risk to health. We use recent data from the UK to explore whether the WHtR-based classification identifies more cardiometabolic risk than the ‘matrix’ based on BMI and waist circumference currently used for screening. Data from the Health Survey for England of 4112 obese people were used to identify cardiometabolic risk, as indicated by elevated glycated Hb, dyslipidemia, and hypertension. HbA1c, total/HDL cholesterol and systolic blood pressure were more strongly associated with WHtR than ‘matrix’. The WHtR 0.5 cut in the initial screening translates to a simple message: the waist should be less than half the height. This allows individuals to be aware of their health risks^3^.

WHtR is a simple anthropometric predictor for central body fat and is easy to use from a health education perspective. WHtR >0.5 was proposed as the first level of health risk. BMI is the most used to define weight status in relation to height, and its units are in kg/m^2^ ^36^. Despite the strong correlation between body fat and BMI, it cannot distinguish between lean mass and fat mass^15^ ^,^ ^35^. Thus, it is important to analyze each factor that makes up MS individually, in order to verify the real impact of bariatric surgery on each comorbidity.

Thus, this study aimed to evaluate the impact of Roux-en-Y gastric bypass comparing the pre- and postoperative period of 1 to 6 months, and the postoperative period of 1 to 2 years in MS and compare waist ratio/height and BMI in relation to the determination of the cardiometabolic risk profile.

## METHOD

Data were collected from the prospective file of electronic medical records of Instituto Paulo Nassif, in Curitiba, PR, from January 2017 to December 2019. This work was approved by the Research Ethics Committee of Mackenzie Evangelical Faculty of Paraná, Curitiba, PR, Brazil, under number 4,324 .990.

### Sample

Eighty patients who participated in a one-year multidisciplinary bariatric surgery preparation program were evaluated.

The inclusion criteria were: 1) patients who underwent bariatric surgery by RYGB and who had laboratory measurements from three different periods; 2) standard collection 1 to 6 months before the operation; 3) standard collection from 1 to 6 months postoperatively; 4) standard collection from 1 to 2 years postoperatively.

The only exclusion criterion was being under 18 years old and over 65 years old.

### Variables analyzed

The following were researched: 1) clinical analyzes were on fasting glucose, serum insulin, glycated hemoglobin, total cholesterol, total triglycerides, HDL and LDL; 2) BMI of each patient before and after the operation in the same periods; 3) abdominal perimeter measured with an inextensible measuring tape in the smallest curvature located between the ribs and the iliac crest, at the normal expiratory moment; 4) waist/height ratio determined by dividing the smallest curvature located between the ribs and the iliac crest, at the normal expiratory moment, by height, measured in centimeters.

### Operative technique

**FIGURE 1:**
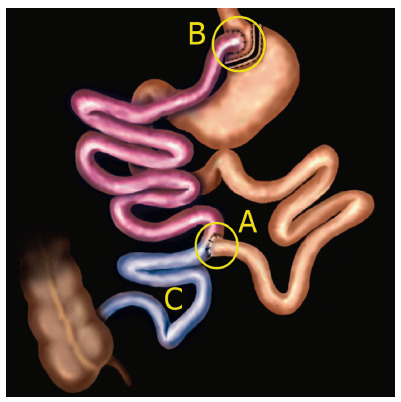
RYGB demonstration: A) Jejunojejunal anastomosis 120 cm from the duodenal flexure (biliopancreatic loop - in brown); B) Gastrojejunal anastomosis 120 cm from the jejunojejunal anastomosis (food loop - in purple); C) Common handle (in blue).

The RYGB consisted of building a small gastric reservoir (stomach with about 20 ml) performing two anastomoses, the gastrojejunal and the jejunojejunal (Figure 1). The rest of the stomach and the diverted intestine were not removed from the body, just excluded from the path taken by food and digestive enzymes. This deviated part anastomoses 120 cm from the duodenojejunal flexure with the jejunum, characterizing the biliopancreatic loop (Figure 1A). From the jejunojejunal anastomosis to the small gastric reservoir, also 120 cm long, characterizes the alimentary loop (Figure 1B). From the jejunojejunal anastomosis to the ileocecal valve, we have the common loop (Figure 1C).

### Statistical analysis

Results of quantitative variables were described as mean, standard deviation, median, minimum and maximum. Categorical variables were described by frequency and percentage. To assess the correlation between two quantitative variables, Pearson’s linear correlation coefficients were estimated. To compare the evaluations before, after 1 to 6 months and after 1 to 2 years, in relation to quantitative variables, the analysis of variance model (ANOVA) with repeated measures and the Bonferroni test were used for comparisons of the two evaluations to two. Regarding categorical variables, comparisons were made using the binomial test. For the comparison of two groups, in relation to quantitative variables, the Student t test for independent samples was used. The condition of normality of continuous variables was assessed using the Kolmogorov-Smirnov test. Data from variables that did not meet this condition were submitted to a logarithmic transformation. Values ​​of p<0.05 indicated statistical significance. For multiple comparisons using the binomial test, p values ​​were corrected by Bonferroni. Data were analyzed using the computer program Stata/SE v.14.1. StataCorpLP, USA.

## RESULTS

### Descriptive statistics

The analysis presented below was performed based on data from 80 patients undergoing RYGB.

**TABLE 1:** Descriptive analysis

Variable	Classif	Result*
Age (years)		41.5±10.4 (20.5 - 66)
Gender	Female	69 (86.3%)
	Male	11 (13.8%)
DM2 (pre)	No	61 (76.3%)
	Yes	19 (23.8%)
Dyslipidemia (pre)	No	38 (47.5%)
	Yes	42(52.5%)
SAH (pre)	No	46 (57.5%)
	Yes	34 (42.5%)

*Described by mean±standard deviation (minimum - maximum) or frequency (percentage); SAH=systemic arterial hypertension

### Evaluation of surgical results on clinical variables

#### Categorical variables

For each of them, the null hypothesis that there was no difference between the distributions in the pre-assessment and in the assessment of 1 to 6 months, vs. the alternative hypothesis that there was. This same comparative analysis was performed for the pre, 1 to 2 years, 1 to 6 months, and 1 to 2 years of evaluation moments. Table 2 presents descriptive statistics of the variables at each time of evaluation and p values of the statistical tests.

**TABLE 2:** Descriptive statistics of the variables DM2, systemic arterial hypertension, and dyslipidemia at each evaluation moment

Variable	Classif	Pre	Post 1 to 6 m	Post 1 to 2 y	p*		
					Pre x post 1 to 6 m	Pre x post 1 to 2 y	Post 1 to 6 m x post 1 to 2 y
DM2	No	61 (76.3%)	78 (97.5%)	78 (97.5%)			
	Yes	19 (23.8%)	2 (2.5%)	2 (2.5%)	<0.001	<0.001	1
SAH	No	46 (57.5%)	73 (91.3%)	75 (93.8%)			
	Yes	34 (42.5%)	7 (8.8%)	5 (6.3%)	<0.001	<0.001	1
Dyslipidemia	No	38 (47.5%)	78 (97.5%)	78 (97.5%)			
	Yes	42 (52.5%)	2 (2.5%)	2 (2.5%)	<0.001	<0.001	1

*Binomial test (p values corrected by Bonferroni); p<0.05; SAH=systemic arterial hypertension

#### Quantitative variables

For each of these variables that met the condition of normality, the null hypothesis was tested that the means in the three assessments (pre, post 1-6 m and post 1-2 y) were equal, vs. the alternative hypothesis that the means were not. In the case of rejection of the null hypothesis, the evaluation moments were compared two by two.

For each of the quantitative variables that did not meet the normality condition, the null hypothesis that the results in the three evaluations (pre, post 1-6 m and post 1-2 y) were equal, vs. the alternative hypothesis that they weren’t. In the case of rejection of the null hypothesis, the evaluation moments were compared two by two.

Table 3 presents descriptive statistics of the variables at each evaluation moment and p values of the statistical tests.

**TABLE 3:** Descriptive statistics of the variables abdominal waist, WHtR, vitamin B12, vitamin D, total cholesterol, HDL, LDL, triglycerides, glycated hemoglobin, insulin, glucose and BMI at each evaluation moment

Variable	Evaluation	Mean ± standard deviation	Median (min-max)	p*			
				Pre x post 1-6 m x pós 1-2 y	Pre x post 1-6 m	Pre x Post 1-2 y	Post 1-6 m x post 1-2 y
AW (cm)	Pre	118.5 ± 9.4	119 (98 - 140)				
	Post 1-6 m	105.2 ± 9.9	106 (81 - 126)				
	Post 1-2 y	90.3 ± 7.7	90 (74 - 110)	<0.001	<0.001	<0.001	<0.001
WHtR	Pre	0.73 ± 0.06	0.72 (0.61 - 0.87)				
	Post 1-6 m	0.65 ± 0.07	0.65 (0.50 - 0.80)				
	Post 1-2 y	0.56 ± 0.05	0.55 (0.45 - 0.69)	<0.001	<0.001	<0.001	<0.001
Vitamin B12	Pre	509.8 ± 276.1	454.5 (209 - 2000)				
	Post 1-6 m	693.5 ± 417.1	591.5 (198 - 2000)				
	Post 1-2 y	639.6 ± 388.5	511 (192 - 2000)	0.002	0.001	0.052	0.733
Vitamin D	Pre	26.8 ± 7.2	26.7 (10.3 - 47.5)				
	Post 1-6 m	31.8 ± 9.3	30.1 (6.9 - 64.3)				
	Post 1-2 y	33.3 ± 10.8	32 (11.3 - 78.4)	<0.001	0.001	<0.001	0.843
Total colesterol	Pre	174.6 ± 34.2	177.5 (66 - 246)				
	Post 1-6 m	149.6 ± 31.8	153 (77 - 217)				
	Post 1-2 y	150.2 ± 28.2	147.5 (94 - 227)	<0.001	<0.001	<0.001	1
HDL	Pre	49.7 ± 12.1	47.5 (21 - 79)				
	Post 1-6 m	45.6 ± 12.0	43.5 (25 - 89)				
	Post 1-2 y	57.5 ± 13.1	56 (28 - 89)	<0.001	0.002	<0.001	<0.001
LDL	Pre	96.6 ± 27.5	100.5 (43 - 169)				
	Post 1-6 m	85.5 ± 27.4	82 (41 - 176)				
	Post 1-2 y	76.3 ± 25.9	74.5 (30 - 166)	<0.001	<0.001	<0.001	0.004
Triglycerides	Pre	152.6 ± 68.3	133 (50 - 423)				
	Post 1-6 m	96.1 ± 34.8	89.5 (41 - 195)				
	Post 1-2 y	82.1 ± 28.7	79.5 (36 - 179)	<0.001	<0.001	<0.001	0.017
Glycated hemog	Pre	6.1 ± 1.6	5.8 (4.8 - 14.4)				
	Post 1-6 m	5.7 ± 1.4	5.3 (4.3 - 13.3)				
	Post 1-2 y	5.6 ± 1.4	5.3 (4.5 - 13.6)	<0.001	<0.001	<0.001	0.367
Insuline	Pre	22.2 ± 12.4	20 (5 - 71.2)				
	Post 1-6 m	8.1 ± 4.4	7 (2 - 24.4)				
	Post 1-2 y	6.2 ± 2.9	5.5 (1 - 16)	<0.001	<0.001	<0.001	0.265
Glucose	Pre	102 ± 25.6	97 (67 - 238)				
	Post 1-6 m	90 ± 16.6	87 (65 - 180)				
	Post 1-2 y	88.3 ± 18.3	84 (71 - 185)	<0.001	<0.001	<0.001	0.504
BMI (kg/m^2^)	Pre	39.8 ± 3.8	39.5 (31 - 52.5)				
	Post 1-6 m	33.2 ± 4.3	33.4 (23.3 - 45.2)				
	Post 1-2 y	26.0 ± 3.2	25.8 (19.6 - 38)	<0.001	<0.001	<0.001	<0.001

*ANOVA with repeated measures and Bonferroni test (post-hoc); p<0.05; AW=abdominal waist; WHtR=waist-to-height ratio; vitamin B12, glycated hemoglobin and glucose data were submitted to logarithmic transformation

### Assessment of the correlation between BMI and WHtR

For each of the evaluation moments (pre, post 1 to 6 m and after 1 to 2 y) and for the differences between the evaluations, the null hypothesis was tested that the correlation coefficient between BMI and WHtR was equal to zero (there was no correlation between the two variables) vs. the alternative hypothesis that the correlation coefficient was non-zero (there was correlation). Table 4 shows the values of Pearson’s linear correlation coefficients and the p values of the statistical tests.

**TABLE 4:** Ratio between waist/height and BMI at each assessment moment

Evaluation	Correlation coefficient of Pearson between relationship WHtR and BMI	p
Pre	0.54	<0.001
Post 1 to 6 m	0.37	0.001
Post 1 to 2 y÷	0.42	<0.001
Reduction (pre - post 1 to 6 m)	0.12	0.294
Reduction (pre - post 1 to 2 y)	0.42	<0.001
Reduction (after 1 to 6 m - after 1 to 2 y)	0.32	0.004

### Assessment of the association between BMI and cardiometabolic risk

For this analysis, two groups were defined according to cardiometabolic risk: group 1 - patients who had cardiometabolic risk in the pre and post 1 to 6 months assessments and stopped having it after 1 to 2 years (n=8); group 2 - patients who had cardiometabolic risk in all assessments (pre, post 1 to 6 m and post 1 to 2 y, n=72)

For each moment of BMI assessment and for the reductions between assessments, the null hypothesis that the BMI means were equal in both groups was tested, vs. the alternative hypothesis that the means were different.

**FIGURE 2:**
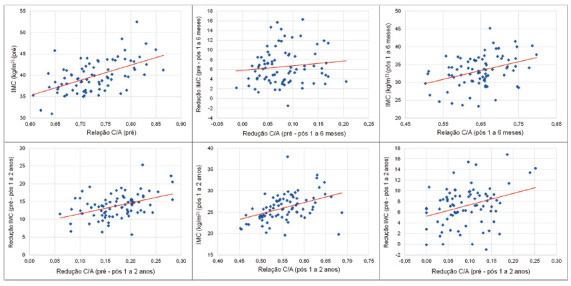
Waist/height ratio (WHtR) and BMI at each assessment time

Table 5 and Figure 2 show descriptive statistics of the evolutionary graphic BMI according to groups 1 and 2, and the p values of the statistical tests. For reductions, positive values indicate a reduction in BMI and negative values indicate an increase.

**TABLE 5:** Descriptive statistics of BMI according to groups 1 and 2

Evaluation	Group	n	BMI (kg/m^2^)		p*
			Mean ± standard deviation	Median (min-max)	
Pre	1	8	38.2 ± 2.7	37.4 (35.3 - 43.5)	
	2	72	40 .0± 3.8	39.7 (31 - 52.5)	0.205
Post 1 to 6 m	1	8	30.9 ± 3.8	31.8 (24.6 - 36.3)	
	2	72	33.5 ± 4.4	33.7 (23.3 - 45.2)	0.116
Post 1 to 2 y	1	8	23.0 ± 1.7	22.9 (21 - 25.8)	
	2	72	26.4 ± 3.2	26.1 (19.6 - 38)	0.004
Reduction (pre - 1 to 6 m)	1	8	7.26 ± 3.83	7.45 (1.3 - 12.6)	
	2	72	6.50 ± 3.67	6.0 (-1.5 - 16.3)	0.578
Reduction (pre - 1 to 2 y)	1	8	15.2 ± 1.69	15.2 (11.9 - 17.7)	
	2	72	13.6 ± 3.56	13.4 (5.7 - 25.3)	0.043
Reduction (after 1 to 6 m - after 1 to 2 y)	1	8	7.91 ± 3.64	7.4 (2.4 - 14.2)	
	2	72	7.11 ± 3.65	7.25 (-1 - 16.8)	0.557

*Student’s t test for independent samples, p<0.05

**FIGURE 3:**
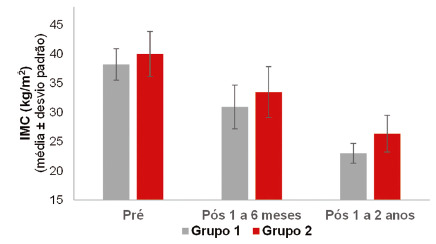
Graph showing the evolution of BMI according to groups 1 and 2

## DISCUSSION

The increased prevalence of MS due to the obesity pandemic, secondary to the current sedentary lifestyle associated with poor diet, has been correlated with higher morbidity and mortality from cardiovascular diseases. MS, in addition to harming the quality of life and health of its individual patients, has become a public health problem. This is due to the costly costs applied to the treatment of chronic diseases involved in MS on an individual basis, in addition to the complications brought about by the syndrome^12^ ^,^ ^13^ ^,^ ^14^.

In this context, metabolic surgery has been shown to significantly improve some of the MS components, the main ones being DM2, dyslipidemia and abdominal perimeter. According to the literature, there is a consensus that there is improvement in the laboratory parameters of patients who undergo metabolic surgery, with RYGB being the most performed procedure currently^18^ ^,^ ^34^.

In addition to clinical and laboratory parameters, a remarkable factor is that the improvement in MS decreases cardiovascular risk. Corroborating other studies^1^ ^,^ ^6^ ^,^ ^9^ ^,^ ^17^ ^,^ ^31^, this research showed improvement in the variables that contribute to the development of cardiovascular diseases.

Metabolic surgery has been shown to have a great anti-diabetogenic effect, which leads to early resolution of the disease picture, even before major weight loss. As proposed by Varaschim et al. (2012)^43^, this fact can be explained by the endocrine effect produced by this procedure. The comparison between the pre- and postoperative periods was significant, while the comparison between the two postoperative periods was not. Similar results were obtained for glycated hemoglobin values, which also decreased when compared pre- and postoperatively.

One of the main factors that contribute to the development of DM2 is insulin resistance, which causes an increase in serum insulin concentration. In this context, metabolic surgery proves to be efficient in decreasing insulin resistance, as the values ​​drop considerably after the procedure. The present study obtained preoperative insulin values averaging 22.2 μIU/ml, in the first postoperative period it was 8.1 μIU/ml and 6.1 μIU/ml in the second - all with significance (p<0.001).

The main risk factor for coronary disease, among the components of MS, is dyslipidemia. It is known that metabolic surgery has a positive influence on improving the lipid profile. As described by Guilbert et al. (2018)^17^ the reduction in triglyceride levels is mainly associated with weight loss, that is, it presents a gradual reduction, a fact that was confirmed by the results of the present study, in which the initial mean was 152.5 mg/dl, the 96 mg/dl in the first postoperative period and 82.1 mg/dl in the second (p<0.001). In the analysis of HDL, the results showed an average of 49.6 mg/dl in the pre, 45.6 mg/dl in the post 1 to 6 months and in the 1 to 2 years period with an average of 57.5 mg/dl - all with significance (p<0.001). Guilbert et al. (2018)^17^ also discuss HDL and explain that, unlike triglycerides, HDL goes through two phases, a decline immediately after the operation and then an increase in the first six months, reflecting the gradual qualitative change, with maturation of the particles, accompanied by an increase in its antioxidant potential, favoring its cardiovascular protection properties. For both components, changes in lifestyle after the operation (increased physical activity and type of diet) have a direct impact ^22^ ^,^ ^33^.

Elevated LDL levels are an important component in the pathophysiology of atherosclerosis; thus, metabolic surgery to improve LDL levels contributes to lower cardiovascular risk. In this work, LDL values were found with a mean of 96.6 mg/dl before the operation, after which the mean dropped to 85.4 mg/dl at first and then to 76.2 mg/dl (p<0.001 in all analyses). The values found corroborate Nassif et al. (2009)^31^, who also showed a progressive drop in LDL. In the analysis of total cholesterol, the results showed a mean of 174.6 mg/dl preoperatively, 149.5 mg/dl and 150.2 mg/dl, respectively, in the first and second postoperative periods. There was significance only in the comparison between pre and postoperative periods (p<0.001).

BMI is the parameter used to indicate the surgical treatment of obesity, in addition to evaluating the efficiency of its short- and long-term results. The variables found were a mean BMI of 39.8 kg/m^2^ preoperatively, which decreased to 33.2 kg/m^2^ in 1 to 6 months after the procedure and to 26 kg/m^2^ in 1 to 2 years - there was significance at all values (p<0.001). Nassif et al. (2009)^31^ showed similar results with a progressive drop in BMI values, which is expected according to the literature. Also, regarding weight loss, Iannelli et al. (2011)^20^ stated that RYGB proved to be significantly efficient.

However, there are several limitations of BMI, which include: non-differentiation of lean and fat mass, making it difficult to assess muscular patients; do not differentiate visceral fat from subcutaneous fat; it has special tables for children and seniors^10^ ^,^ ^26^ ^,^ ^30^.

Kornerup, et al. (2019)^24^ reported a high risk of developing micronutrient deficiencies due to extensive changes in the anatomy and physiology of the gastrointestinal tract in RYGB. In 2016, the American Society of Bariatric and Metabolic Surgery updated its nutritional guidelines aimed at bariatric patients, which described great variability in vitamin deficiencies, both pre and postoperatively. The prevalence of preoperative vitamin deficiencies of 30% for vitamin B12 and 90% for vitamin D was then identified. In the present study, the mean of vitamin B12 in the analyzed periods was within the normal range, ranging from 509.8 to 639.6. These 80 patients studied were rigorously followed up and supplemented both pre- and post-operatively. Johnson et al., (2019)^23^ demonstrated that postoperative deficiencies had a prevalence of up to 20% for vitamin B12 and 100% for vitamin D. Our results showed preoperative vitamin D deficiency with a mean of 26.8.

RYGB compromises vitamin B12 absorption because almost no gastric acid remains in the gastric pouch, and as a result, food-bound release of B12 is substantially decreased. In addition, the production of intrinsic factor - a protein derived from the parietal cell necessary for the intestinal absorption of B12 - is reduced or absent in the bypassed stomach. Furthermore, B12 malabsorption is enhanced by the late introduction of pancreatic enzymes in the distal jejunum^24^ ^,^ ^42^ ^,^ ^45^.

As described, vitamin D deficiency is the most common preoperative deficiency, and is related to insufficient sun exposure and reduced hepatic hydroxylation^7^ ^,^ ^15^ ^,^ ^16^. To prevent postsurgical vitamin D deficiency, oral vitamin D supplementation of 800 IU daily is generally recommended by the American Association of Clinical Endocrinologists and The Obesity Society^25^.

Recent researches with the aim of determining limit values ​​for WHtR in different populations indicated that a cutoff point of 0.5 is the most indicated value for both genders, all ages and different populations^5^ ^,^ ^27^ ^,^ ^35^. Several studies have shown that WHtR is also a better indicator for the health of children and adolescents than other anthropometric indicators. And the cutoff point of 0.5, which has been proposed, is close to those recommended for adults^21^ ^,^ ^29^ ^,^ ^44^.

Lima et al. (2010)^26^ verified the existence of a common waist-to-height ratio in male individuals, aged between 18 and 25 years, with normal fat percentages, to provide a personalized and non-generalized method of measuring waist circumference. The analysis of the sample of 174 individuals resulted to be in the age group of 21.2±2.1 years, with height of 174.3±6.2 cm, with a percentage of fat of 10.8%, with measurement of the abdominal circumference of 75.5±5.7 cm, and with the waist/height ratio presenting the value of 0.43±0.033. They concluded that there is a common relationship between the waist-to-height ratio among men aged between 18 and 25 years with a normal fat percentage of 43% of their height.

Study by Lucas et al. (2020)^28^, using the cutoff point of 0.5 in WHtR as a reference, evaluated the development of an equation that could determine the appropriate waist measurement of the smallest abdominal perimeter, also having as premise the WHtR, but in a sample 454 individuals, 249 males and 205 females, between 18 and 65 years old, without the state of obesity. Regarding the percentage of height as a measure of the smallest waist circumference, the total female sample had an average of 44.2±1.1% and the male 45.3%±1.5. For women this percentage determined the equation of the waist-to-height ratio represented by X=(age+217)/5.875, and for men X= (age+190.89) /5.2222. The “X” in the equations represents the percentage of height measurement so that the individual fits into the category of adequate in relation to percentages of fat and BMI.

Our study found that the WHtR values ​​after two years of postoperative RYGB were equivalent to 0.56, with a reduction equivalent to 60.7% if the objective was to reach 0.45 in the study by Lucas et al. (2020)^27^. 

The method of analysis of the waist/height ratio (WHtR) takes into account a perimetric measure that presents a predictive profile of the quantitative situation involving visceral fat. Therefore, the characteristics of the local subcutaneous adipose tissue may make a difference in this interpretation.

We justify the score above 0.5 due to the anatomophysiological condition common to individuals in this obesity scenario, as in addition to hypertrophy, subcutaneous adipocyte hyperplasia is present, and ends up determining a functional residue, linked to intense weight loss. These residues are called “functional and aesthetic bodily sequelae”, as mentioned by Cintra Junior et al.^8^

Such changes correspond to what is conventionally called dysmorphia, characterized by dermofat accumulations predominantly in the arms, breasts, abdomen and thigh, which is corroborated by the large number of patients in the RYGB postoperative period requiring submission to cosmetic surgeries, notably abdominoplasty ( dermolipectomy), as mentioned by Baroudi and Moraes^4^.

The sum of the metabolic results presented indicates that this score is compatible with a low amount of body fat after 02 years of postoperative BYGR. Therefore, for this select group of patients, we believe that this anatomical and functional aspect should be taken into account, as a future criterion for reinterpreting the cutoff point, and promoting comparison with the post-surgical esthetic state. In this sense, we can infer that there was a greater reduction in the parameters of the waist/height ratio than the BMI in this period.

Although both did not manage to reach the level of normality according to their scales, when the WHtR was correlated with the BMI and the other MS markers, the superiority of the former for the identification of the cardiometabolic risk profile was evident.

## CONCLUSION

The RYGB improves the metabolic syndrome and the waist/height ratio (WHtR) is superior to the BMI in the assessment of the cardiometabolic risk profile.
